# A novel *TLE6* mutation, c.541+1G>A, identified using whole‐exome sequencing in a Chinese family with female infertility

**DOI:** 10.1002/mgg3.1743

**Published:** 2021-07-15

**Authors:** Bin Mao, Xueling Jia, Hongfang Liu, Xiaojuan Xu, Xiaodong Zhao, Yue Yuan, Hongxing Li, Xiaoling Ma, Lili Zhang

**Affiliations:** ^1^ The Reproductive Medicine Hospital of the First Hospital of Lanzhou University Lanzhou Gansu China

**Keywords:** female infertility, ICSI, IVF, mutation, *TLE6* mutation

## Abstract

**Background:**

Oocytes have a lot of maternal RNAs and proteins, which are used by the early embryo before zygotic genome activation. Transducin‐like enhancer of split 6 (TLE6) is a component of a subcortical maternal complex which plays a critical role in early embryonic development.

**Methods:**

The patient had been diagnosed with primary infertility for 6 years and had undergone multiple failed in vitro fertilization (IVF)/intracytoplasmic sperm injection (ICSI) cycles. Genomic DNA samples were extracted from her parents’ peripheral blood as well as hers. Whole‐exome sequencing and Sanger validation were performed to identify candidate variants.

**Results:**

We identified a novel transducin‐like enhancer of split 6 (*TLE6*) gene mutations in the female patient with recurrent IVF/ICSI failure. The patient carried a homozygous mutation (NM_001143986.1(*TLE6*): c.541+1G>A) and had viable but low‐quality embryos. Her parents both had heterozygous mutations at this locus.

**Conclusion:**

Our study expands the mutational and phenotypic spectrum of *TLE6* and suggests the important role of *TLE6* during embryonic development. Our findings have implications for the genetic diagnosis of female infertility with recurrent IVF/ICSI failure.

## Graphical abstract

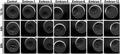

## 1 INTRODUCTION

Transducin‐like enhancer of split (TLE) proteins are the mammalian homologs of Groucho, a conserved family of co‐repressors present in animals. The Groucho family has six main member genes in mammals (*TLE1*‐*6*) [OMIM: *TLE1*(*600189), *TLE2*(*601041), *TLE3*(*600190), *TLE4*(*605132), *TLE5*(*600188), *TLE6* (*612399)]. *TLE1*‐*4* are full‐length genes, *TLE5* and *TLE6* are two short‐type genes that can inhibit the function of *TLE1*‐*4* genes, and there is a close relationship between the short‐type genes and full‐length genes. In a study of human cerebellar granule neurons, TLE5 inhibited the *TLE1*‐mediated anti‐apoptotic effect and promoted cell apoptosis (Zhang et al., 2008). The transducin‐like enhancer of split 6 (TLE6) [OMIM: #612399] is a member of the TLE transcriptional co‐repressor family. *TLE6* was first identified by Dang et al as the target of hepatic leukemia factor (E2a) and is widely expressed in mouse embryos and adult tissues (Dang et al., 2001). In addition to its short length, its sequence is very different from other Groucho/Tle family members. The mouse TLE6 protein contains 581 amino acids with a molecular weight of 65 kDa. Unlike typical TLE1‐4 proteins, the work of Li and Roberts (2001) showed that TLE6 has only five tryptophan‐aspartic acid (WD) repeat units, missing domains that are involved in the Groucho/TLE oligomerization, in binding to specific transcription factors (N‐terminal of TLE), and that are involved in the interaction of most transcription factors (C‐terminal WD40 repeat domain) (Marcal et al., 2005). As most of the N‐terminal domains are missing, TLE6 may not be able to form heterodimers with other *TLE* genes and may interact with other proteins alone.

The *TLE6* gene is highly expressed in the ovaries of newborns, and the TLE6 protein combines with a variety of maternal effector proteins to form the maternal effector complex (subcortical maternal complex), which regulates early embryonic development and cell division in mice and sheep, is necessary for the early embryonic development of mammals (Bebbere et al., 2014; Li et al., 2008). The TLE6 protein controls the symmetrical division of fertilized mouse eggs by regulating the actin cytoskeleton. Knocking out TLE6 causes infertility in mice (Yu et al., 2014) and humans (Alazami et al., 2015). So far, only one variant has been documented in Human Gene Mutation Database (HGMD) (http://www.hgmd.cf.ac.uk/ac/gene.php?gene=TLE6) and three pathogenic/likely pathogenic variants reported in ClinVar (https://www.ncbi.nlm.nih.gov/clinvar). With the development of massively parallel exon sequencing, the number of diagnosed cases of *TLE6*‐related female infertility will increase. Here we report one female patient with primary infertility for 6 years and had undergone multiple failed in vitro fertilization (IVF)/intracytoplasmic sperm injection (ICSI) cycles due to a splice‐site mutation in *TLE6* (NM_001143986.1(*TLE6*): c.541+1G>A). Our results provide more evidence of TLE6 function in human embryo development.

## 2 METHODS

### 2.1 Ethical compliance

The study and procedures were approved by the Research Ethics Committee of First Hospital of Lanzhou University. The subjects gave informed signed consents.

### 2.2 Genomic DNA extraction

Genomic DNA samples of the patient (diagnosed in the First Hospital of Lanzhou University) and her family members were isolated from peripheral blood using a HiPure Blood DNA Mini Kit (Magen). The DNA concentration and purity were measured with a NanoDrop 1000 spectrophotometer (Thermo Scientific).

### 2.3 Sequence analysis

Whole‐exome capture was carried out using the BGI Exome V4 (59 Mb) Kit (BGI), and the sequencing was performed with MGISEQ‐2000 sequencers (BGI). Sequences were aligned to the human genome assembly GRCh37, and variants were annotated. The frequency of corresponding mutations was determined using the Exome Aggregation Consortium (ExAC) database (http://exac.broadinstitute.org/), and the functional effects of the mutations were predicted with the in silico algorithms PolyPhen‐2 (http://genetics.bwh.harvard.edu/pph2/) and PROVEAN (http://provean.jcvi.org). The variants were prioritized based on the following filtering criteria: (i) a frequency below 0.1% for homozygous variants or below 1% for compound heterozygous variants in the ExAC database, (ii) loss of function alleles or damaging missense variants predicted by PolyPhen‐2 or PROVEAN, (iii) variants with high gene expression in human oocytes and embryos, and (iv) variants with an embryogenesis‐related function. Homozygosity mapping was performed with HomozygosityMapper for affected individuals from the family to determine the existence of candidate homozygous variants. Three tools (HSF (http://umd.be/Redirect.html), MaxEnt (http://hollywood.mit.edu/burgelab/maxent/Xmaxentscan_scoreseq.html), and SpliceAI (https://github.com/Illumina/SpliceAI)) were used to predict splice‐site mutation effect on splicing. The candidate variants were then confirmed by Sanger sequencing in patient and her parents. The primers used for confirming *TLE6* (NM_001143986.1: c.541+1G>A) were 5′‐CCTGCAGGAGTCGAGCTTTGAG‐3′ and 5′‐CACCCCTCTGGATAGAGCTGAG‐3′ and the product length was 369 bp.

## 3 RESULTS

### 3.1 Clinical characteristics of the affected individuals

We discovered a novel *TLE6* mutation in the infertile woman with recurrent failure of IVF/ICSI attempts. The affected individual had been diagnosed with primary infertility for 6 years and had undergone multiple failed IVF/ICSI cycles. Her husband exhibited normal sperm counts, motility, and morphological features. The patient's clinical information is summarized in Table 1.

**TABLE 1:** Oocyte and embryo characteristics of the IVF and ICSI attempts of the female patient

Age (years)	Infertility duration (years)	Fallopian tube examination	IVF/ICSI attempts	Oocytes retrieved	Fertilized oocytes	Viable embryos on day 3	Embryos transferred	Outcomes
32	6	The right fallopian tube was patent, and the left fallopian tube was not smooth	1st IVF	11	10	7 (IV embryos)	0	Embryonic arrest
			2nd ICSI	20	8	8 (IV embryos)	0	Embryonic arrest

Abbreviations: ICSI, intracytoplasmic sperm injection; IVF, in vitro fertilization.

**FIGURE 1:**
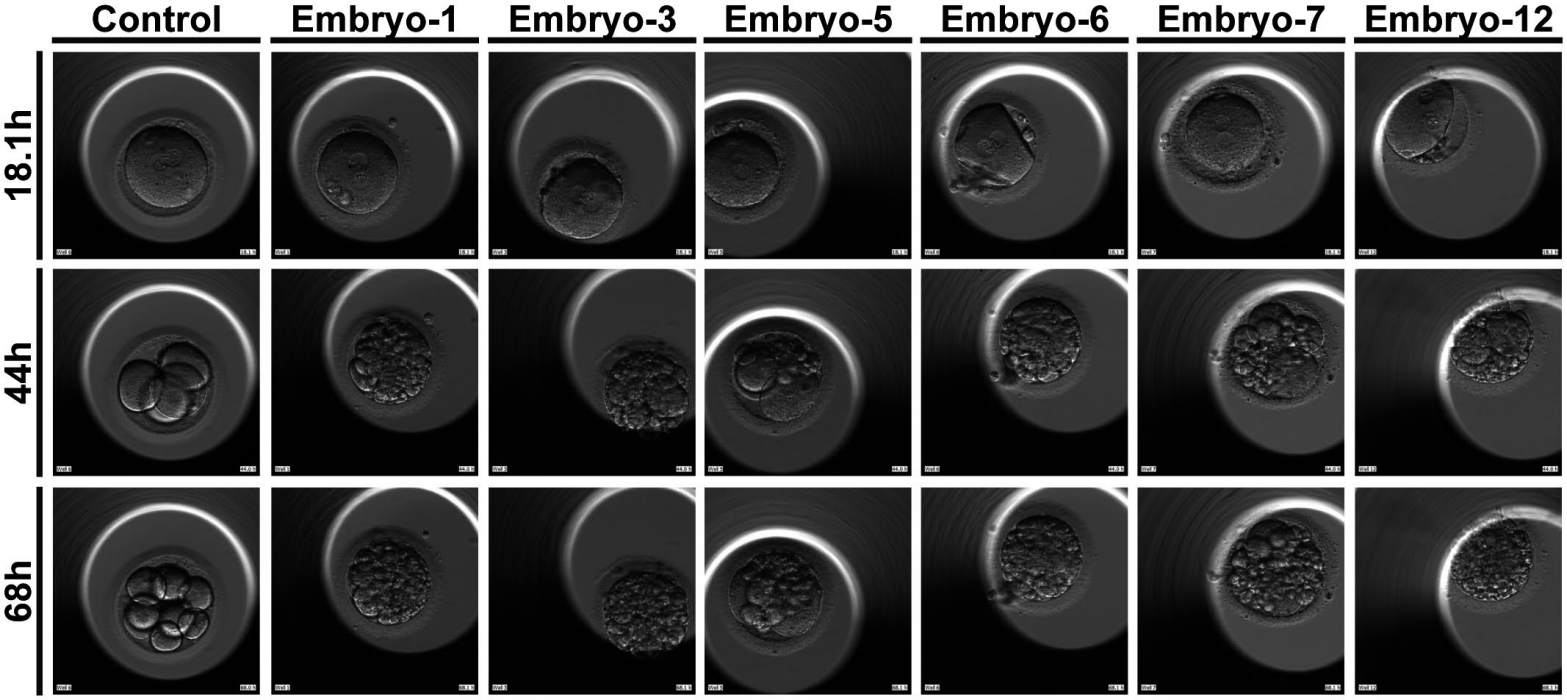
Phenotypic features of the patient's embryos. The morphologies of the embryos from a control and the patient were photographed under a light microscope on day 1, day 2, and day 3 after fertilization

The 32‐year‐old proband female had two failed IVF/ICSI attempts. In the first IVF cycle, 11 oocytes (9 MII, 1 AMII and 1 MI) were retrieved and 5/10 of them displayed abnormal fertilization (5 0PN (pronucleu) zygotes and 5 2PN zygotes). Finally, all the seven embryos were grade IV and stopped developing on day 3. In the second ICSI cycle, 20 oocytes (15 MII and 5 MI) were retrieved and eight 2PN zygotes were obtained after ICSI but all of them also stopped developing on day 3 (Figure 1). Altogether, 31 oocytes were retrieved, and finally, there were 15 viable grade IV embryos. All the embryos were embryonic arrest with fragmentation >50% and none of them could be transferred (Table 1).

### 3.2 Identification of mutations in TLE6

**FIGURE 2:**
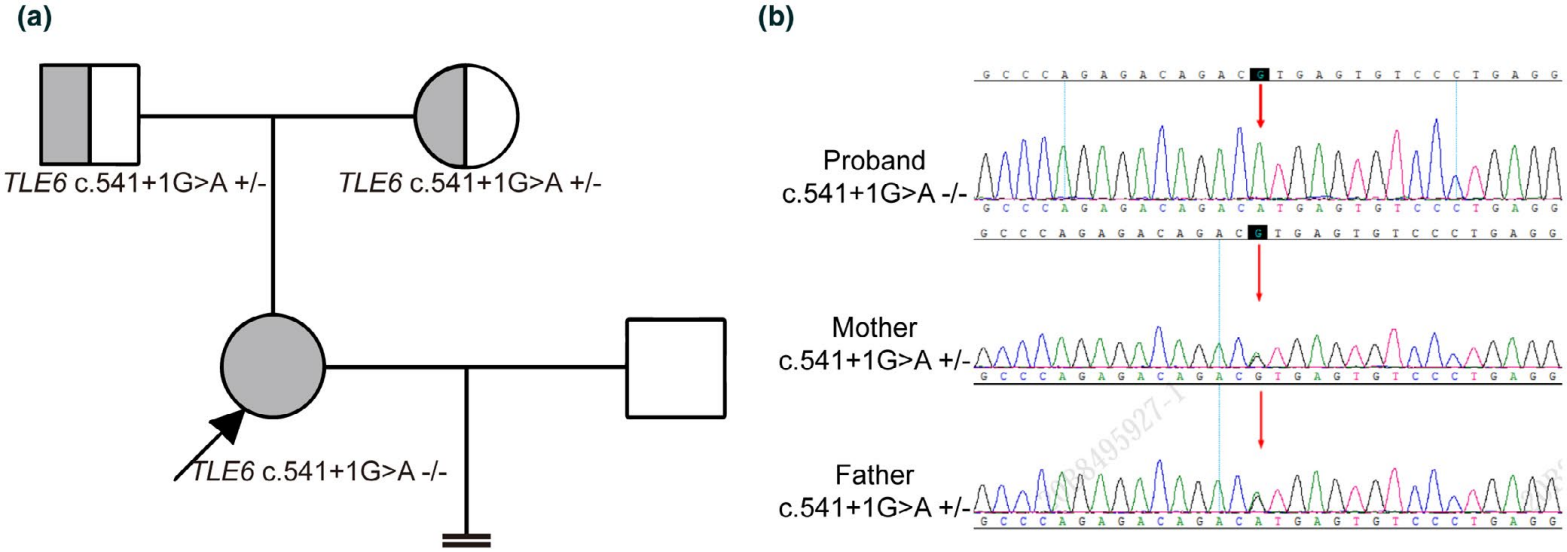
Identification of *TLE6* mutation. (a) Pedigree of the patient family carrying *TLE6* mutations that lead to embryonic arrest. Squares denote males and circles denote females, filled symbols represent patients with primary infertility, semi‐filled symbols represent *TLE* mutation carriers and open symbols represent unaffected individuals. (b) Sanger sequencing confirmation of *TLE6* c.541+1G>A mutation in proband and her parents

A homozygous mutation in intron seven of *TLE6* (NM_001143986.1: c.541+1G>A) was identified in the proband. This mutation was verified by Sanger sequencing, and both of her parents carried a heterozygous *TLE6* mutation, indicating a recessive inheritance pattern (Figure 2a,b). This mutation was a classical mutation type which would disrupt RNA splicing. The prediction results of three different tools (HSF, MaxEnt, and SpliceAI) also showed that the alteration of the donor site (NM_001143986.1: c.541+1G>A) most probably affected splicing (Supplementary Materials). This mutation has not been reported in these databases, including ClinVar, HGMD, gnomAD, ExAC, dbSNP, and 1000G, and suggesting a novel mutation.

## 4 DISCUSSION

The literature showed that the *TLE6* gene may affect the ability of cell proliferation, differentiation, and cycle. With the discovery and extensive research into the core inhibitors of TLE in humans and animals, there is a consensus in the literature that TLE‐mediated inhibitory effects are modulated by spatial and temporal distribution. TLE proteins do not bind directly to DNA; they interact with several DNA‐binding proteins, thereby inhibiting the expression of downstream genes and proteins, and regulating life activities, neuronal differentiation, and tumorigenesis in invertebrates and vertebrates (Chen et al., 2008; Marcal et al., 2005; Verginelli et al., 2013). In cortical neural progenitor cell, the TLE6 can interfere with cell differentiation into neuron (Marcal et al., 2005). In colon cancer cell, TLE6 interacts with the gastrointestinal tumor suppressor RUNX3, increasing tumor cell proliferation, colony formation, cell migration, and xenograft tumorigenesis (Chen et al., 2008).

*Tle6* has been reported that it is expressed in various tissues of animals (Hoffman et al., 2008), and the expression of *Tle6* in ovarian tissue greatly affects the development of early embryos in mammals (Bebbere et al., 2014; Duncan et al., 2014; Li et al., 2008; Zhu et al., 2015). Tle6 is essential for the growth and development of cells. The work of Yu et al showed that, most of the fertilized eggs of Tle6‐mutant female mice cannot cleave, and thus caused infertility in the transition from early embryos to two cells in mice (Yu et al., 2014). Studies have indicated that female patients with recurrent miscarriages may have mutations in the *TLE6* gene, and the phenotype is like Tle6‐knockout mice. However, so far, only one case has been reported in the HGMD database, and only three pathogenic/ likely pathogenic mutation types (c.805_806del (p.Lys269fs), c.1133del (p.Ala378fs), c.1529C>A (p.Ser510Tyr)) have been included in the ClinVar database (https://www.ncbi.nlm.nih.gov/clinvar/?term=tle6%5Bgene%5D).

As we found, *TLE6* mutant embryos (c.541+1G>A) showed abnormalities already by the second day of development and all the embryos were arrested on day 3 with fragmentation >50%, the work of Alazami et al also indicated that TLE6 did not affect the discharge of mature oocytes from patients, it affected the termination of embryo cleavage at cleavage stage (Alazami et al., 2015). When Tle6 was knocked out in mouse spermatogonia, although the cell growth did not appear to stagnate, the growth rate was extremely low. Although we did not confirm the mRNA isoform expression in the proband, different bioinformatic tools (HSF, MaxEnt, and SpliceAI) all suggested that this variant would affect splicing. The exon adjacent to this splice‐site is not in the key domain of TLE6 protein (such as WD40 repeat containing domain), so this variant (*TLE6* c.541+1G>A) might cause abnormal protein function by forming a new splice‐ site affecting the spatial structure of the protein.

In summary, our study identified a novel mutation in *TLE6* associated with early embryonic arrest and thus expanded the mutational spectrum of *TLE6* and the phenotypic spectrum of patients with such mutations. Our findings add new information on the genetic basis of female infertility and suggest that *TLE6* might be a therapeutic target as well as genetic diagnostic marker for recurrent IVF/ICSI failure.

## CONFLICT OF INTEREST

The authors declare that they have no conflict of interest.

## AUTHORS CONTRIBUTIONS

Bin Mao and Xueling Jia contributed to conception and design; Xiaoling Ma and Lili Zhang were responsible for providing guidance and advice on the project; Yue Yuan and Hongxing Li were responsible for clinical information collection; Bin Mao, Hongfang Liu, Xiaojuan Xu, and Xiaodong Zhao were responsible for experiments and data analysis, and Bin Mao was responsible for manuscript writing and revision. All authors contributed to acquisition, revised manuscript, and agreed to be accountable for all aspects of work ensuring integrity and accuracy. All authors read and approved the final manuscript.

## CONSENT FOR PUBLICATION

Consent for publication was obtained from the patient and her parents.

## Supporting information

Supplementary Material

## Data Availability

The data that support the findings of this study are available from the corresponding author upon reasonable request.
