# Supplementary material for: A novel TLE6 mutation, c.541+1G>A, identified using whole‐exome sequencing in a Chinese family with female infertility
Source: Mol Genet Genomic Med. 2021 Jul 15;9(8):e1743. doi: 10.1002/mgg3.1743 (PMC8404233; doi:10.1002/mgg3.1743)
Supplement: Supplementary file 1 — Supplementary Material [file MGG3-9-e1743-s001.docx]

**Supplementary Materials**

# HSF and MaxEnt prediction results

HSF (<http://umd.be/Redirect.html>);

MaxEnt (<http://hollywood.mit.edu/burgelab/maxent/Xmaxentscan_scoreseq.html>)


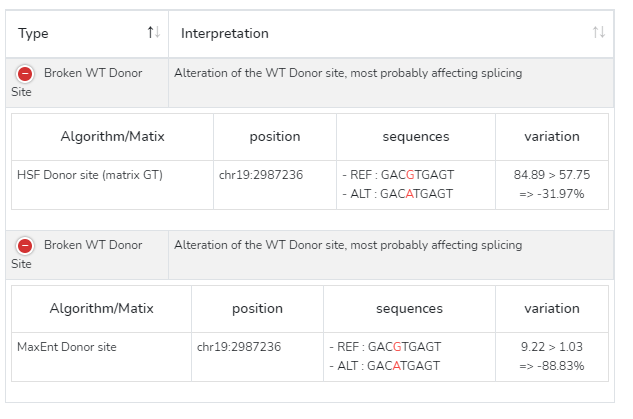


*(Reference genome GRCh38)*

For HSF prediction, strong splice-sites always present a CV higher than 80 and less strong splice-sites with a CV ranging between 70 and 80. Only a minor fraction of active sites show a CV between 65 and 70. A ‘broken’ splice-site is defined as an above-threshold site and the difference in scores is <-10%. So, 57.75 (with -31.97% difference in score) for chr19: 2987236(2987237-1) (NM_001143986.1: c.541+1G>A) means it would affect splicing.

For MaxEnt prediction, values range from -20 to +20 and the threshold is 3. A ‘broken’ splice site is defined as an above-threshold site and the difference in scores is < -30%. So, 1.03 (with -88.83% difference in score) for chr19: 2987236(2987237-1) (NM_001143986.1: c.541+1G>A) means it would affect splicing.

# 2.SpliceAI prediction results

SpliceAI (<https://github.com/Illumina/SpliceAI>)

| **ID** | | | **Description** | | | | | | | |  |
| --- | --- | --- | --- | --- | --- | --- | --- | --- | --- | --- | --- |
| ALLELE | | | Alternate allele | | | | | | | |  |
| SYMBOL | | | Gene symbol | | | | | | | |  |
| DS_AG | | | Delta score (acceptor gain) | | | | | | | |  |
| DS_AL | | | Delta score (acceptor loss) | | | | | | | |  |
| DS_DG | | | Delta score (donor gain) | | | | | | | |  |
| DS_DL | | | Delta score (donor loss) | | | | | | | |  |
| DP_AG | | | Delta position (acceptor gain) | | | | | | | |  |
| DP_AL | | | Delta position (acceptor loss) | | | | | | | |  |
| DP_DG | | | Delta position (donor gain) | | | | | | | |  |
| DP_DL | | | Delta position (donor loss) | | | | | | | |  |
| **ALLELE** | **SYMBOL** | **DS_AG** | | **DS_AL** | **DS_DG** | **DS_DL** | **DP_AG** | **DP_AL** | **DP_DG** | **DP_DL** | |
| A | TLE6 | 0 | | 0 | 0.23 | 0.96 | 15 | -28 | -20 | -1 | |

For SpliceAI prediction, Delta score of a variant, defined as the maximum of (DS_AG, DS_AL, DS_DG, DS_DL), ranges from 0 to 1 and can be interpreted as the probability of the variant being splice-altering (0.2 (high recall/ likely pathogenic), 0.5 (recommended/ pathogenic), and 0.8 (high precision/ pathogenic)). Delta position conveys information about the location where splicing changes relative to the variant position (positive values are downstream of the variant, negative values are upstream)

The probability that the position chr19: 2987236(2987237-1) (NM_001143986.1: c.541+1G>A) is used as a splice donor decreases by **0.96**. So, the alteration of the donor site (NM_001143986.1: c.541+1G>A) most probably affects splicing.
